# *Achyranthes bidentata* Polypeptide Protects Schwann Cells From Apoptosis in Hydrogen Peroxide-Induced Oxidative Stress

**DOI:** 10.3389/fnins.2018.00868

**Published:** 2018-11-30

**Authors:** Meiyuan Li, Ye Zhu, Wenqiang Peng, Hongkui Wang, Ying Yuan, Xiaosong Gu

**Affiliations:** ^1^School of Biology and Basic Medical Sciences, Soochow University, Suzhou, China; ^2^Key Laboratory of Neuroregeneration of Jiangsu Province and Ministry of Education, Co-Innovatioin Center of Neuroregeneration, Nantong University, Nantong, China

**Keywords:** *Achyranthes bidentata* polypeptides, schwann cells, oxidative stress, cell apoptosis, hydrogen peroxide

## Abstract

ABPPk, the active ingredient separated from *Achyranthes bidentata* polypeptides, is a traditional Chinese medicine with multiple pharmaceutical properties. In this study, we investigated the molecular mechanisms of ABPPk in protecting Schwann cells (SCs) from H_2_O_2_-induced cell apoptosis. The viability of SCs pretreated with ABPPk was elevated significantly by MTT assay estimation. Meanwhile, the apoptosis of SCs was reduced which was showed in flow cytometry and transferase-mediated dUTP nick end labeling analysis. Furthermore, the addition of ABPPk also increased the activities of SOD and GSH accompanied with a decrease in MDA and LDH activities. According to Western blot analysis, the upregulation of Bcl-2, also downregulation of Bax and cleaved caspase-3 were demonstrated in SCs which was ABPPk pretreated. Further research showed that PI3K/AKT and ERK1/2 pathways in SCs have been activated after pretreatment of ABPPk. Collectively, results in our study suggested that ABPPk protected SCs from H_2_O_2_-induced oxidative damage by reducing the expression of apoptotic molecules and enhancing the activities of antioxidant enzymes, which inhibited the apoptosis of SCs modulated by PI3K/AKT and ERK1/2 signaling pathways. In our perspectives, ABPPk as an active factor with its antioxidative activities has potential and promising therapeutic effects in the prevention of neurologic disorders.

## Introduction

Schwann cells (SCs), an important component of the peripheral nervous system (PNS) that wrapping both myelinated and unmyelinated nerve fibers, could excrete a large number of growth factors to promote axonal growth and myelinization (Jessen and Mirsky, 1997; Woszczycka-Korczynska et al., 2013). Damage to SCs is likely to induce cell apoptosis and restrict functional recovery of peripheral nerves (Zhao et al., 2017). It is reported in previous studies that apoptosis of SCs caused by oxidative stress is the common and vital mechanism of peripheral neuropathy (Purves et al., 2001). Oxidative stress-induced cell apoptosis is implicated as an important pathogenic factor in many neurodegenerative diseases (Olanow, 1993; Behl, 1999; Finkel and Holbrook, 2000; Choi et al., 2006). H_2_O_2_ known as a precursor of reactive oxygen species (ROS) (Sherer et al., 2002), can cause dysfunction of DNA synthesis, protein expression, and mitochondria structure, and also complete disruption of cellular integrity and cell function (Pizarro et al., 2009; Fernandez-Checa et al., 2010; Wang C.H. et al., 2013). Accordingly, inhibition of the oxidative damage to SCs will improve the potential ability of protection from damage and regenerative efficacy of peripheral nerve injuries.

*Achyranthes bidentata*, listed in the Chinese Pharmacopoeia, is a significant medicinal plant with multiple therapeutic effects, such as anti-inflammatory, antipyretic, and diuretic activities (He et al., 2014). In previous research, we found that *A. bidentata* polypeptides (ABPP) that isolated from the aqueous extract of *A. bidentata* Blume possesses a protective effect on *N*-methyl-D-aspartate (NMDA)-induced apoptosis of hippocampal neurons (Shen et al., 2008) and could promote peripheral nerve regeneration in rats and rabbits (Yuan et al., 2010; Wang Y. et al., 2013; Cheng et al., 2014). Furthermore, ABPPk purified by HPLC, exhibits excellent neuron-protective efficiency (Yu et al., 2014; Peng et al., 2018). However, little is known about the mechanism by which ABPPk exerts its protective role on SCs, especially in the aspect of oxidative stress. Consequently, the aim of this research is to validate the hypothesis whether ABPPk has a cytoprotective effect on SCs against oxidative stress damage *in vitro* and to reveal its underlying molecular mechanism.

In our study, we used H_2_O_2_-induced cell apoptosis models to investigate the potential cytoprotective activity of ABPPk on SCs. Results revealed that ABPPk treatment significantly protects SCs from apoptosis in oxidative stress induced by H_2_O_2_. Nevertheless, the beneficial effects of ABPPk against oxidative stress damage likely coupled with the PI3K/AKT and ERK1/2 signaling pathways. It is suggested that ABPPk treatment is potentially a useful intervention of preventing SCs against oxidative stress-induced cell apoptosis.

## Materials and Methods

### Preparation of ABPPk

ABPP was extracted from *A. bidentata* Blume, which was obtained from a Chinese medicine grocery, identified by Professor Haoru Zhao from China Pharmaceutical University (Peng et al., 2018). HPLC was further used to purify ABPP and the preparation protocol of ABPPk was previously described (Cheng et al., 2014; Yu et al., 2014).

### Cell Treatment

SCs were cultured from the sciatic nerves of 1-day-old SD rats as previously described (Mantuano et al., 2008; Yu et al., 2012). In brief, to remove fibroblasts, anti-Thy1.1 antibody (Sigma, St. Louis, MO, United States) and rabbit complement (Invitrogen, Carlsbad, CA, United States) were further added to SCs isolated. Then SCs was cultured in Dulbecco’s modified Eagle’s medium (DMEM) supplemented with 10% fetal bovine serum in a humidified atmosphere of 5% CO_2_ and 95% air at 37°C (Li et al., 2015). Primary cultured SCs were identified by immunostaining and flow cytometric analysis (Dubovy et al., 2001; Shen et al., 2012). H_2_O_2_ was used to establish the apoptosis model as previously reported (Luo et al., 2012). H_2_O_2_ was freshly diluted from 30% H_2_O_2_ stock solution with DMEM medium to a 400 μM final concentration prior to each experiment. To determine the effect of ABPPk on H_2_O_2_-exposed SCs, SCs were pretreated with ABPPk for 12 h followed by co-treatment of ABPPk with H_2_O_2_ for 24 h. In a single experiment, each treatment was performed in triplicate.

### Cell Viability Assay

Cell viability was determined by using MTT assay. SCs onto 96-well plates were treated differently with a density of 2 × 10^5^cells/ml. Then 20% sodium dodecyl sulfide (SDS) was added to dissolve the resulting formazan, following the incubation of SCs with MTT (0.5mg/ml) for 4 h at 37°C, which was described by measuring the absorbance (OD) values at 570 nm using Microplate reader (BioTek, United States) (Shen et al., 2011).

### Tunel Staining of Apoptosis Cells

The SCs were seeded at a density of 2 × 10^5^cells/ml to experience different treatments. The terminal deoxynucleotidyl transferase-mediated dUTP nick end labeling (TUNEL) assay by the DeadEndTM Fluorometric Tunel system (Promega, Madison, WI, United States) was showed according to manufacturer’s instructions to detect the apoptotic cells. Differential interference contrast microscopy images were then obtained randomly (Peng et al., 2018). The ratio of Hoechst positive cells and Tunel positive cells were respectively calculated and at least five images of each sample in different visions were observed (Geoffroy et al., 2017).

### Flow Cytometry

The purity of primary cultured SCs was further evaluated by flow cytometric analysis with S100 antibody (He et al., 2012; Shen et al., 2012). And the extent of apoptosis was measured through AnnexinV-FITC apoptosis detection kit (BD Bioscience, San Jose, CA, United States) on the basis of the manufacturer’s instruction. In brief, the SCs were resuspended in 1× Binding Buffer and stained with FITC-Annexin V and propidium iodide (PI) for 15 min at room temperature followed by washing twice with cold PBS. The apoptotic cells were analyzed by flow cytometry (BD Biosciences) (Zhou et al., 2015). The relative ratio of early apoptotic cells was calculated which was repeated more than three times.

### LDH Release Assay

As an indicator of cell injury, LDH was released into the cell culture supernatant when cells undergo apoptosis (Chien et al., 2013). The cell viability was estimated by the amount of LDH release using LDH Detection Kit according to the manufacturer’s protocol (Li et al., 2014). The absorbance of samples was surveyed at 450 nm under a microplate reader (BioTek, United States).

### Measurements of Intracellular SOD, MDA, and GSH Contents

SCs cells were seeded in 6-well plates and grown overnight. Next, being exposed to H_2_O_2_ and ABPPk, the cellular SOD, MDA, and GSH were assessed by following the manufacturer’s instructions of assay kits as described previously (Li et al., 2014). The activity of SOD was detected at 550 nm with an ELx-800 microplate reader. The MDA contents in each cultured SCs were measured at a wavelength of 532 nm. The absorbance of GSH was measured at 405 nm by using a microplate reader (BioTek, United States).

### Immunofluorescence Staining

The SCs were seeded in 24-well dishes. After treatment and fixation, the cells were incubated with anti-cleaved caspase-3 (1:1000; Abcam) or anti-S100 (1:1000; Abcam) primary antibody solution at 4°C overnight, and then, secondary antibodies (Donkey anti-Mouse IgG-Alex-488, 1:200; Goat anti-Rabbit IgG-Cy3, 1:200; Invitrogen) were added for 1 h at room temperature. Finally, the nuclei were stained with DAPI or Hoechst 33342. The reaction was observed and photographed under a fluorescence microscopy (AxioImager M2, Zeiss).

### Western Blotting Analysis

The protein concentration was determined using the Micro BCA Protein Assay Kit (Pierce, Rockford, IL, United States) (Yi et al., 2016). Equal amounts of protein samples were separated on 10% SDS-PAGE and transferred to polyvinylidene fluoride membranes (Millipore, Bedford, MA, United States) (Gu et al., 2015). The membranes were blocked in 5% nonfat dry milk for 2 h and incubated with primary antibody anti-Bcl-2 (1:1000; Abcam), anti-Bax (1:1000; Abcam), anti-caspase-3 (1:1000; Abcam), anti-cleaved caspase-3 (1:1000; Abcam), anti-phospho ERK1/2 (1:1000; Abcam), anti-ERK1/2 (1:1000; Abcam), anti-PI3K (1:1000; Abcam), anti-phospho PI3K (1:1000; Abcam), anti-phospho AKT (1:1000; Abcam), anti-AKT (1:1000;Abcam), and anti-GAPDH (1:5000; Abcam) overnight at 4°C. After three washes with Tris-buffered saline with 0.1% Tween-20 (TBST), HRP-conjugated secondary antibodies (Pierce) were used to incubate the membranes for 1 h at room temperature. The membranes were developed using a chemiluminescence reagent (Roche) after three times washed in TBST and exposed to Kodak exposure films. GAPDH served as an internal control in order to make normalization and relative quantitative analysis of target protein expression (Feng et al., 2016).

### Statistical Analysis

All data are presented as mean ± SEM. All experiments were undertaken in triplicates. Statistical significance was conducted by one-way analysis of variance (ANOVA) with Bonferroni’s *post hoc* test. A value of *P* < 0.05 was considered to be statistically significant.

## Results

### ABPPk Protected SCs Against H_2_O_2_-Induced Cell Cytotoxicity

**FIGURE 1:**
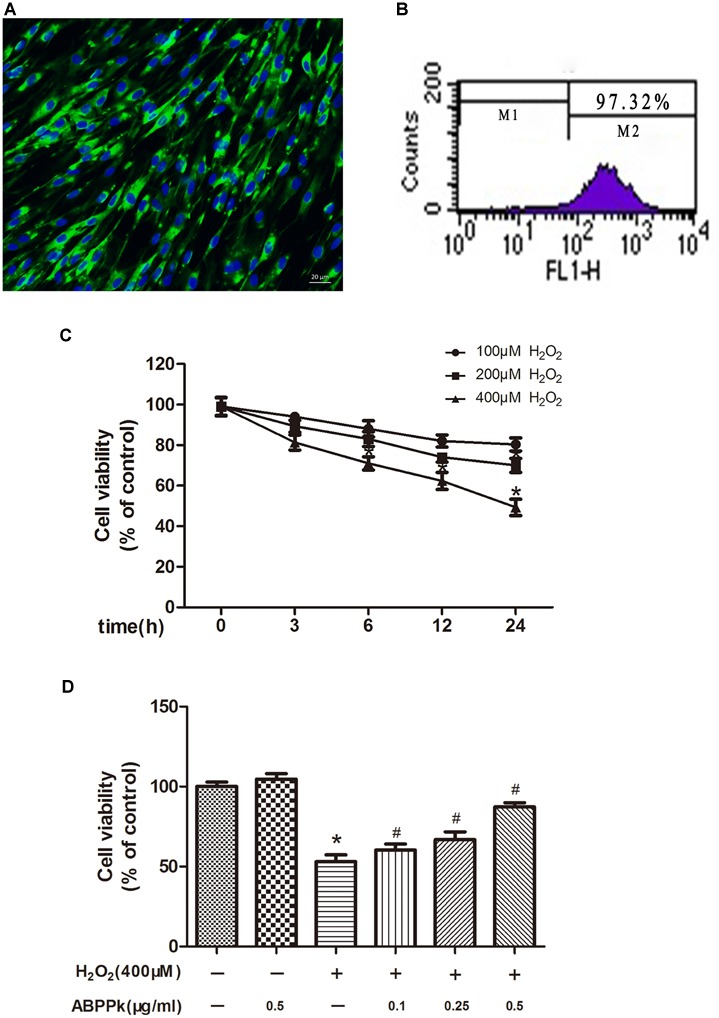
Effect of ABPPk on viability in H_2_O_2_-treated SCs. **(A)** Characterization of primary cultured SCs with S100 (green) immunocytochemistry combined with Hoechst 33342 staining (blue). **(B)** Representative flow cytometric analysis data illustrate 97.32% of S100-positive cells (M2). **(C)** SCs were induced by 100, 200, and 400 μM H_2_O_2_ for the indicated times (3, 6, 12, and 24 h). **(D)** SCs were pre-incubated with ABPPk (0.1, 0.25, and 0.5 μg/ml) for 12 h and then exposed to 400 μM H_2_O_2_. ^∗^*P* < 0.05 vs. the control cells. ^#^*P* < 0.05 vs. the cells treated with H_2_O_2_ alone. Scale bar = 20 μm.

The purity of primary cultured SCs was confirmed by immunocytochemistry with anti-S100 antibody (Figure 1A) and flow cytometry data (Figure 1B), which indicated that 97.32% of the cell population was S100-positive. The MTT results showed that H_2_O_2_ stimulation (100, 200, and 400 μM) could gradually reduce cell activity in a time- and dose-dependent manner. As shown in Figure 1C, an obvious decrease of viability was observed at 24 h with 400 μM H_2_O_2_ exposure (53.1 ± 4.9%, compared to the control). To evaluate the potential cytoprotective effect of ABPPk against oxidative stress-induced SCs death, SCs were pretreated with ABPPk at various concentrations (0.1, 0.25, and 0.5 μg/ml) for 12 h followed by exposure to H_2_O_2_ at a final concentration of 400 μM for 24 h. After pretreatment with ABPPk, the cell viability was significantly increased in a dose-dependent manner compared with H_2_O_2_ treatment alone. Specifically, treatment with ABPPk prior to H_2_O_2_ exposure at different concentrations (0.1, 0.25, and 0.5 μg/ml) increased the survival to 60.3 ± 2.2, 66.8 ± 3.2, and 87.3 ± 1.1%, respectively (*P* < 0.01) (Figure 1D). These data suggest that ABPPk protected SCs against oxidative stress in SCs. In addition, ABPPk at these concentrations was not significantly cytotoxic.

### ABPPk Protected SCs Against H_2_O_2_-Induced Cell Apoptosis

**FIGURE 2:**
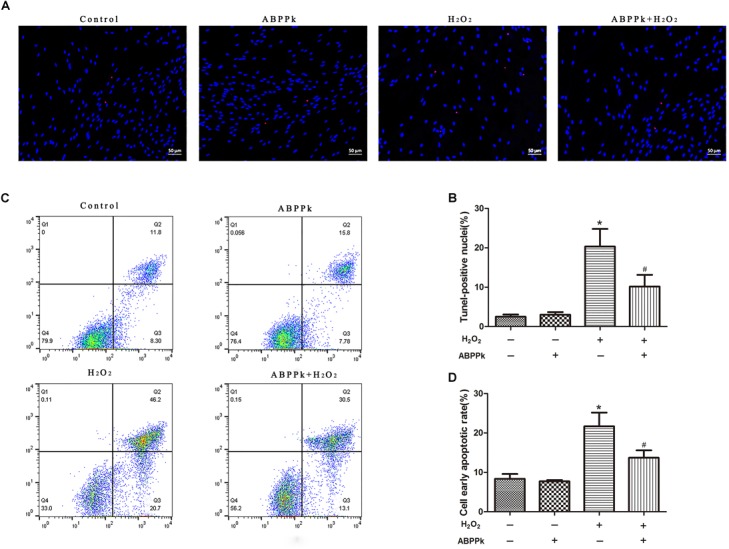
Effects of ABPPk on H_2_O_2_-induced cell apoptosis. SCs were pretreated with ABPPk for 12 h and then exposed to H_2_O_2_ for additional 24 h. **(A)** Tunel-positive cells (red) indicate apoptotic cells and Hoechst 33342 (blue) was used to stain the nuclei. **(C)** The PI/AnnexinV-FITC staining and flow cytometry results show the ratio of cell apoptosis, the lower right panel which describes the early apoptotic cells. The percentage of TUNEL-positive cells and the rate of early apoptosis are shown by two histograms **(B,D)**, respectively. The results are expressed as the mean ± SEM, *n* = 5. ^∗^*P* < 0.05 vs. the control cells. ^#^*P* < 0.05 vs. the cells treated with H_2_O_2_ alone. Scale bar = 50 μm.

TUNEL analysis indicated that ABPPk prevented cultured SCs from H_2_O_2_-induced apoptosis. The TUNEL-positive cells were significantly increased from 3.04 ± 1.02 (control) to 21.86 ± 1.86% by exposure to H_2_O_2_ alone (*P* < 0.01). After pretreatment with ABPPk (0.5 μg/ml) for 12 h, H_2_O_2_ stimulation was added and the number of TUNEL-positive cells significantly reduced to 10.58 ± 4.49% as compared to H_2_O_2_ stimulation alone (*P* < 0.01) (Figures 2A,B). The apoptotic rate was also quantitatively analyzed by flow cytometry with AnnexinV-FITC/PI staining and provided further evidence that ABPPk pretreatment prevented cultured SCs from H_2_O_2_-induced apoptosis. The results of the apoptotic cell rate showed that ABPPk inhibited the early SCs apoptotic rate induced by H_2_O_2_ (Figures 2C,D). The ability of ABPPk to inhibit the SCs apoptosis under H_2_O_2_ conditions was exhibited according to the above results.

### ABPPk Inhibits Apoptosis as Measured by Bax, Bcl-2, and Cleaved Caspase-3 in SCs

**FIGURE 3:**
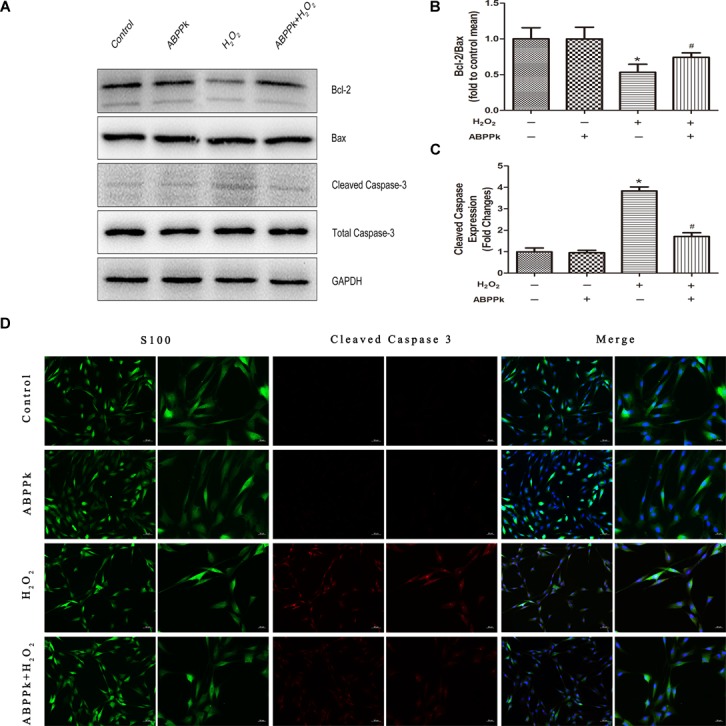
ABPPk inhibits apoptosis as measured by Bax, Bcl-2, and cleaved caspase-3 in SCs. The SCs were pretreated with 0.5 μg/ml of ABPPk and then exposed to 400 μM H_2_O_2_ to induce cell apoptosis. **(A)** The protein levels of Bax, Bcl-2, and cleaved caspase-3 were measured by Western blot with the antibodies is shown. GAPDH was used as a loading control. **(B,C)** Histograms show Western blot analysis data and cleaved caspase-3 expression data, respectively. **(D)** Caspase activation in H_2_O_2_-treated SCs assessed by cleaved caspase-3 immunocytochemistry. As shown in the images, the absence and presence of ABPPk was marked with S100 (green), DAPI (blue), and cleaved caspase-3 (red) staining. The results are expressed as the mean ± SEM, *n* = 5. ^∗^*P* < 0.05 vs. the control cells. ^#^*P* < 0.05 vs. the cells treated with H_2_O_2_ alone. Scale bar = 50 μm (low magnification) and 20 μm (high magnification).

To confirm ABPPk protection against H_2_O_2_-induced apoptosis, apoptosis-associated protein (Bcl-2, Bax, and cleaved caspase-3) levels were measured. Western blot analyses indicated that treatment with ABPPk significantly increased the expression of Bcl-2 and significantly decreased the expression of Bax and cleaved caspase-3, compared with that of H_2_O_2_ treatment alone (Figures 3A–C). Immunofluorescence staining was performed to detect the activation of caspase-3, and cleaved caspase-3 induced by H_2_O_2_, while fewer ABPPk-treated cells displayed activated caspase-3 (Figure 3D).

### ABPPk Attenuates H_2_O_2_-Induced Oxidative Injury

**FIGURE 4:**
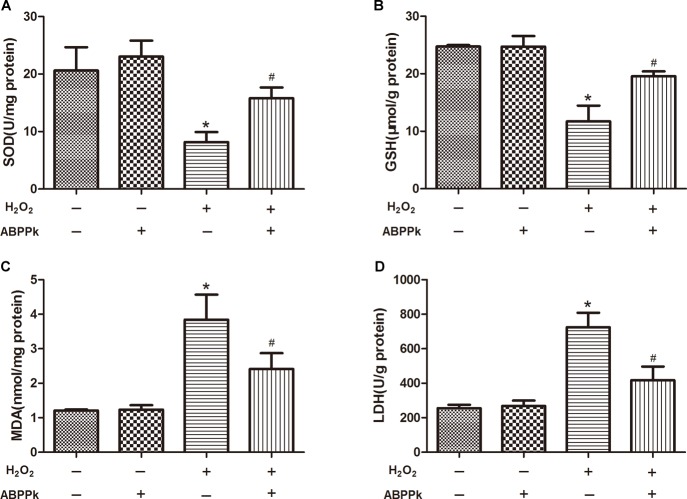
The effects of ABPPk on the intracellular SOD, MDA, and GSH levels and LDH released by H_2_O_2_-injured SCs. **(A)** The SOD level of SCs H_2_O_2_ treated with the addition of ABPPk or not. **(B)** The intracellular GSH level of SCs treated with H_2_O_2_ in the absence or presence of ABPPk. **(C)** The intracellular MDA level of SCs treated with H_2_O_2_ in the absence or presence of ABPPk. **(D)** The release of LDH from SCs damaged by H_2_O_2_ with or without protection of ABPPk. The results are expressed as the mean ± SEM, *n* = 6. ^∗^*P* < 0.05 vs. the control cells. ^#^*P* < 0.05 vs. the cells treated with H_2_O_2_ alone.

More and more evidence has shown that H_2_O_2_-induced oxidative stress and apoptosis plays the crucial role in SCs dysfunction and death. SOD and GSH levels were decreased due to the treatment of the cells by adding H_2_O_2_ along for 24 h (*P* < 0.01, compared to the control group). However, co-incubation with ABPPk remarkably attenuated the changes in the content of SOD and GSH (*P* < 0.01) (Figures 4A,B) when compared to H_2_O_2_-treated cells. Additionally, treating with H_2_O_2_ for 24 h, the intracellular MDA and LDH release were increased (*P* < 0.01, compared to the control group), however, incubation with ABPPk produced a significant decrease in the intracellular level of MDA and LDH compared with the H_2_O_2_ group (*P* < 0.01) (Figures 4C,D).

### PI3K/AKT and ERK1/2 Pathways Are Involved in ABPPk Treatment Suppressing H_2_O_2_ Induced Apoptosis in SCs

**FIGURE 5:**
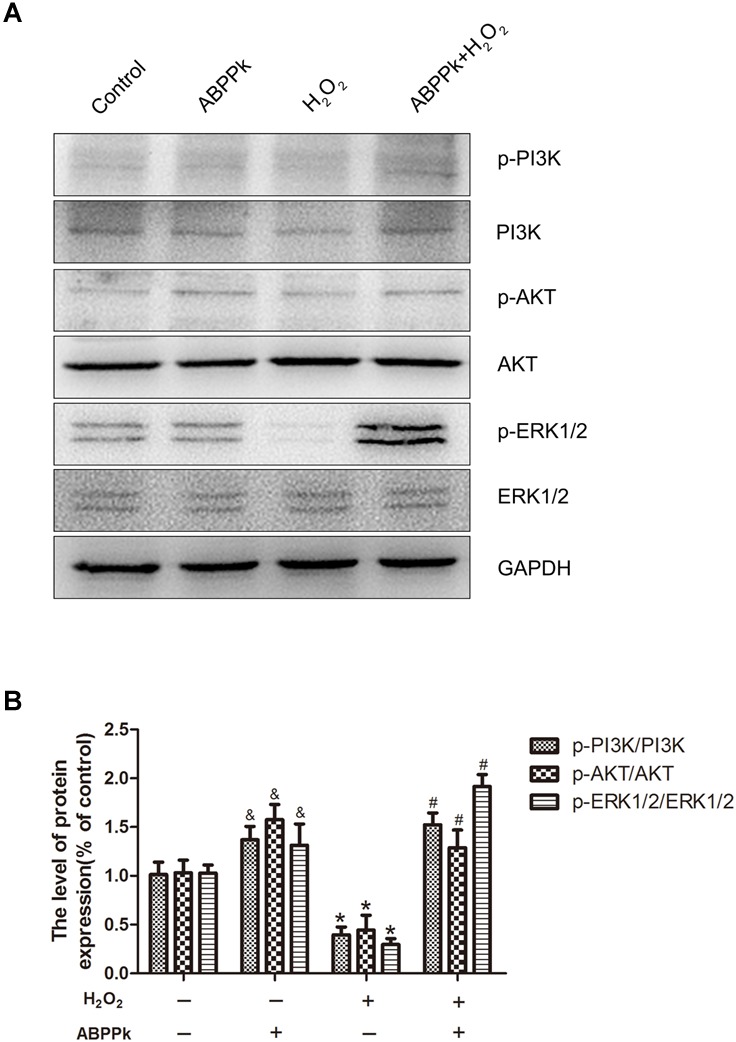
ABPPk treatment activates the PI3K/AKT and ERK1/2 pathways in SCs. **(A)** SCs were treated with 400 μM H_2_O_2_ with or without 0.5 μg/ml ABPPk. The cells were collected and analyzed by Western blot for the expression of p-PI3K and PI3K, p-AKT and AKT, and p-ERK1/2 and ERK1/2. **(B)** Histogram shows data of p-PI3K/PI3K, p-AKT/AKT, and p-ERK/ERK levels from Western blot analyses. GAPDH was used as the protein loading control and band density normalization. ABPPk vs. control: ^&^*P* < 0.05; H_2_O_2_ vs. control: ^∗^*P* < 0.05; ABPPk + H_2_O_2_ vs H_2_O_2_: ^#^*P* < 0.05, *n* = 3.

Here, the expression levels of p-PI3K, PI3K, p-AKT, AKT, p-ERK1/2, and ERK1/2 were analyzed by Western blot. Compared with the control group, the p-PI3K/PI3K, p-AKT/AKT, and p-ERK1/2/ERK2 ratios were significantly reduced in the H_2_O_2_ group at 24 h. ABPPk treatment significantly reversed the decreased expression levels of p-PI3K/PI3K, p-AKT, and p-ERK1/2 in SCs exposed to H_2_O_2_ for 24 h (Figures 5A,B). These data suggest that ABPPk might suppress H_2_O_2_-induced apoptosis through the PI3K/AKT and ERK1/2 signaling pathways.

**FIGURE 6:**
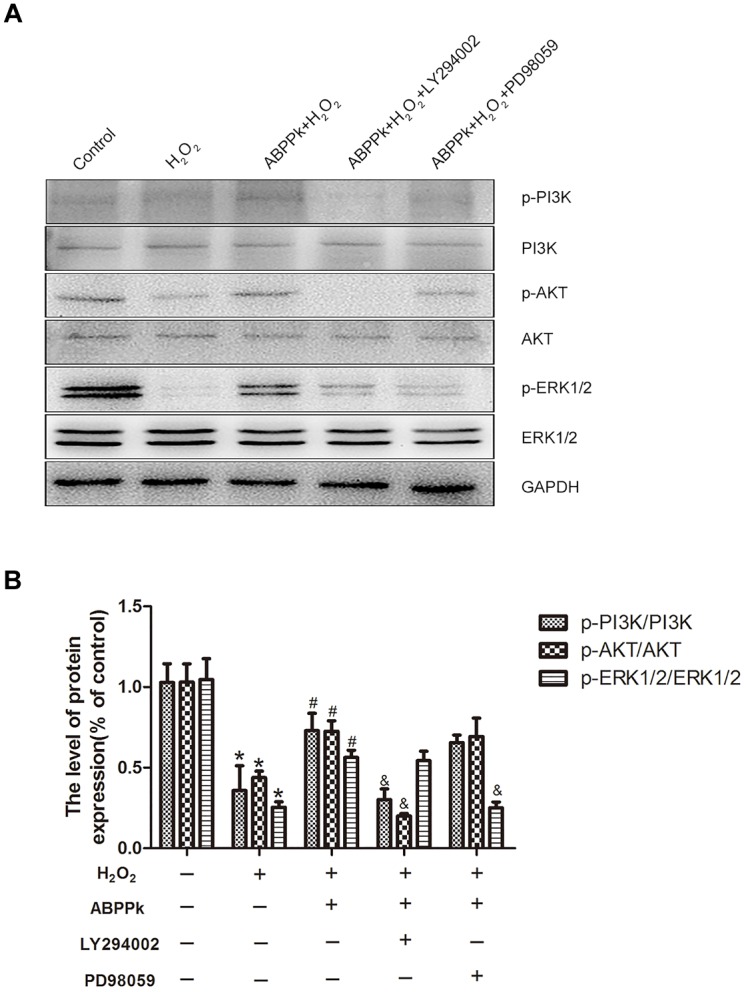
Effects of the LY294002 and PD98059, PI3K inhibitor, and ERK1/2 inhibitor, on oxidative damage attenuated by ABPPk in SCs. **(A)** Western blot shows the p-PI3K/PI3K, p-AKT/AKT, and p-ERK/ERK in SCs pretreated with LY294002 (10 μM) and PD98059 (10 μM) for 30 min before the treatment of H_2_O_2_ (400 μM) and ABPPk (0.5 μg/ml) for 24 h. **(B)** Densitometric analyses illustrates the results of p-PI3K/PI3K, p-AKT/AKT, and p-ERK/ERK. H_2_O_2_ vs control: ^∗^*P* < 0.05; ABPPk + H_2_O_2_ vs H_2_O_2_: ^#^*P* < 0.05; ABPPk + H_2_O_2_ + LY294002 or ABPPk + H_2_O_2_ + PD98059 vs ABPPk + H_2_O_2_: ^&^*P* < 0.05, *n* = 3.

To further confirm our presumption that the role of ABPPk in the inhibition of H_2_O_2_-induced oxidative stress was related to the activation of PI3K/AKT and ERK1/2 pathways, two signal inhibitors, LY294002 and PD98059 (10 μM), respectively, were added to SCs. We found that the increased levels of p-PI3K, p-AKT, and p-ERK1/2 by ABPPk treatment were decreased after inhibitor treatment (Figures 6A,B). The agonists of these two signaling pathways had been performed (Supplementary Figure S1).

**FIGURE 7:**
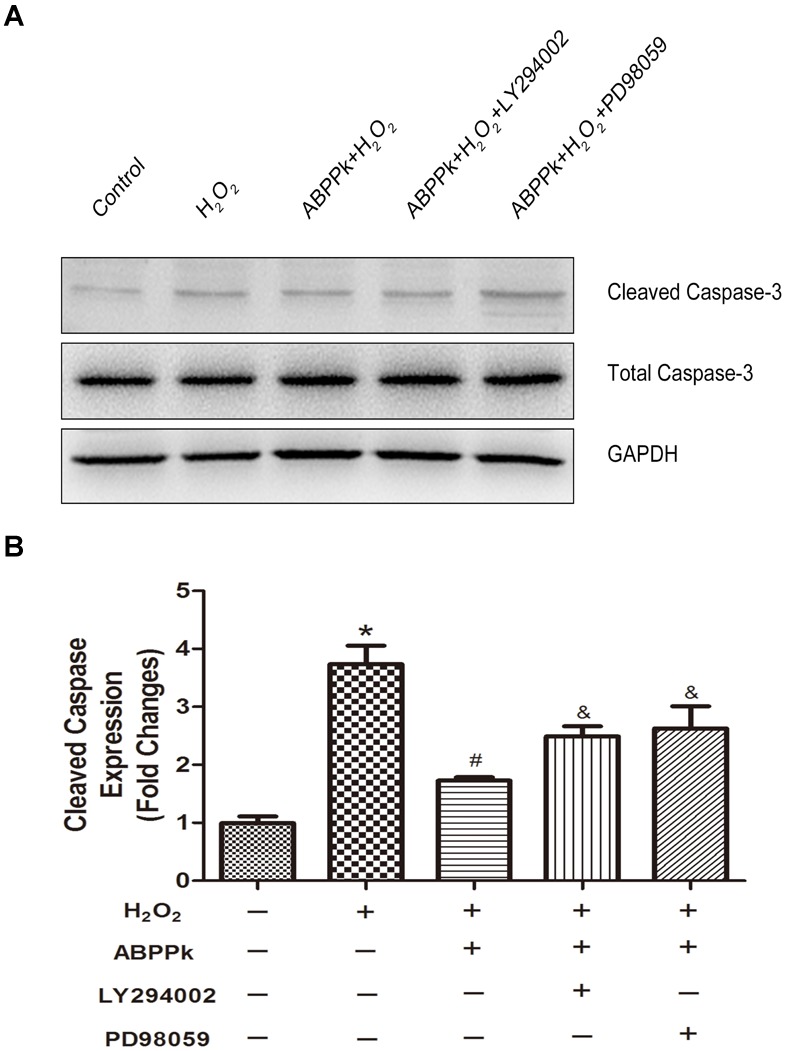
The protective ABPPk on apoptosis induced by H_2_O_2_ are partly impaired through the inhibition of the PI3K/AKT and ERK1/2 pathways. **(A)** Protein expression levels of cleaved caspase-3. **(B)** Quantification data of apoptotic markers cleaved caspase-3 in each group. H_2_O_2_ vs control: ^∗^*P* < 0.05; ABPPk + H_2_O_2_ vs H_2_O_2_: ^#^*P* < 0.05; ABPPk + H_2_O_2_ + LY294002 or ABPPk + H_2_O_2_ + PD98059 vs ABPPk + H_2_O_2_: ^&^*P* < 0.05, *n* = 3.

In terms of the caspase-dependent pattern in our cell model and ABPPk was proved to reduce the caspase-3 activation (Figure 3), we further evaluate if LY294002 and PD98059 could affect the protection of ABPPk according to the change in the protein level of cleaved caspase-3. As described in Western blot, ABPPk treatment significantly prevented the H_2_O_2_ induced upregulation of cleaved caspase-3 in SCs, which was eliminated after the addition of the inhibitor LY294002 or PD98059 (Figures 7A,B). It was suggested that ABPPk plays a protective role on the cell apoptosis induced by H_2_O_2_ *via* the PI3K/AKT and ERK1/2 signaling pathways on the basis of the results.

## Discussion

In this study, H_2_O_2_ was used to establish an oxidative damage model of SCs. The experimental results demonstrated that ABPPk markedly protects SCs against H_2_O_2_-induced apoptosis, which may be due to the activation of PI3K/AKT and ERK1/2 signaling pathways. In addition, ABPPk could increase SOD and GSH activity and decrease MDA and LDH level in oxidation damaged SCs. Furthermore, ABPPk inhibited the apoptosis of SCs by increasing the level of Bcl-2 and decreasing the levels of Bax and cleaved caspase-3. In accordance with the results, ABPPk has a potent protective effect on peripheral nerve injuries by protecting SCs from oxidative stress-induced apoptosis.

*Achyranthes bidentata* was extensively investigated based on its multiple physiological functions, including anti-inflammatory, antirheumatic, antipyretic, and diuretic effects. A large amount of previous studies have discussed the effectiveness of ABPP in nervous system (Shen et al., 2008, 2013). Recently, we separated the ABPP by HPLC and then one fraction was obtained, which was named ABPPk. It exhibits excellent neuron-protective efficiency (Cheng et al., 2014; Yu et al., 2014). In this research, we have found that ABPPk could protect SCs from oxidative stress-induced damage. It is the first time that the protective roles of ABPPk on SCs in oxidative stress were investigated.

Such neurodegenerative disorders, such as Alzheimer’s disease, Parkinson’s disease, and the like attribute to oxidative stress that induces cell apoptosis are characterized by overloading ROS (Roy et al., 2007; Numakawa et al., 2011; Kurian et al., 2017). Oxidative injuries to SCs bring about demyelination and slow formation of axon, which might play the important role in PNS injury and regeneration. H_2_O_2_ is widely used as an oxidant for studies *in vitro*. Previous research showed that H_2_O_2_ injury was able to induce cell apoptosis with concentration and time dependence (Wang and Huang, 2005; Kaji et al., 2010; Bai et al., 2012). In the present experiment, it is confirmed that SCs treated with 400 μM H_2_O_2_ exhibits loss of cell viability. Pretreatment with 0.1–0.5 μg/ml ABPPk, however, significantly attenuate the loss of cell viability induced by H_2_O_2_ in a dose-dependent manner.

The apoptosis process could be regulated by proteins of Bcl-2 family through balancing of pro-apoptotic (Bax) and anti-apoptotic (Bcl-2) products (Bone, 1991; Jezek and Plecita-Hlavata, 2009). In view of the results, ABPPk decreased H_2_O_2_-induced apoptosis of SCs by upregulation of Bcl-2 as well as downregulation of Bax. In addition, caspase 3 acts as an executor in the protease cascade reactions of cell apoptosis (Mao et al., 2007; Hua et al., 2015). The recent work suggested that caspase-3 is important for cell morphology and biochemical events related to the process of apoptosis (Makin, 2016; Zhang et al., 2016). In our study, we confirmed that the cultured SCs showed a remarkable increase of cleaved caspase-3 after exposed to H_2_O_2_ and pretreatment with ABPPk significantly attenuated activation of caspase-3, which suggest that the potential anti-apoptotic effects of ABPPk against H_2_O_2_-induced cell apoptosis of SCs which might be regulated by the apoptosis-related caspase-3 protein expression and activation.

During the process of cell apoptosis, the MDA, as a byproduct induced by excessive ROS, is considered to be an indicator for oxidative stress (Wang et al., 2012). SOD and GSH, as antioxidants for the prevention of free-radical damage caused by ROS, provide a repair mechanism for oxidative stress (Wang et al., 2012). In our study, SOD and GSH were decreased after exposure to H_2_O_2_, indicating the impairment of SCs in oxidative stress. Moreover, the MDA level has an obvious elevation associated with an increase of LDH release. Nonetheless, these H_2_O_2_-induced cellular events were considerably blocked when SCs were co-cultured with ABPPk. The results reveal that the increase of endogenous antioxidant preservation may represent a potential mechanism of ABPPk for attenuating intracellular oxidative stress.

PI3K/AKT and ERK1/2 signaling, as we know, is vital to cell survival and cell apoptosis under stress exposure (Gerrits et al., 2006; Chen et al., 2015). In this research, the expression levels of p-PI3K, p-AKT, and p-ERK1/2 were reduced under the exposure to H_2_O_2_ and this situation was partially reversed by the ABPPk treatment. It shows that ABPPk activate the PI3K/AKT and ERK1/2 pathways in SCs resulting in the suppression of effect of H_2_O_2_ stress induced-apoptosis of SCs. In accordance with the results, the effects of pharmacological inhibitors for PI3K/AKT (LY294002) and ERK1/2 (PD98059) in SCs, and the reduction of cell death by ABPPk depressed by inhibitor treatment were demonstrated.

In summary, our study indicates that ABPPk could protect SCs from H_2_O_2_-induced oxidative stress and cell apoptosis, as well as activation of PI3K/AKT and ERK1/2 signaling pathways. The results lay a ground work for deeper understanding of the molecular mechanism of ABPPk-regulated anti-apoptotic activity in SCs and also provide valuable information of new strategies for better clinical treatments of peripheral nerve injuries.

## Author Contributions

XG conceived the research and provided ABPPk. ML designed and performed the experiments and analyzed data. YZ, WP, and YY performed the experiments. ML and HW wrote and revised the manuscript. All authors have read and approved the final manuscript.

## Conflict of Interest Statement

The authors declare that the research was conducted in the absence of any commercial or financial relationships that could be construed as a potential conflict of interest.

## Supplementary Material

FIGURE S1 Effects of the PI3K/AKT agonists 1,3-Dicaffeoylquinic acid and ERK1/2 agonists Honokiol on ABPPk induced attenuation of oxidative damage. **(A)** SCs were treated with the agonists 1,3-Dicaffeoylquinic acid (10 μM) and Honokiol (10 μM) for 1 h before the treatment of H_2_O_2_ (400 μM) and ABPPk (0.5 μg/ml) for 24 h. **(B)** Densitometric analyses illustrates the results of p-AKT/AKT and p-ERK/ERK. H_2_O_2_ vs control: ^∗^*P* < 0.05; ABPPk + H_2_O_2_ vs H_2_O_2_: ^#^*P* < 0.05; ABPPk + H_2_O_2_ + 1,3-Dicaffeoylquinic acid or ABPPk + H_2_O_2_ + Honokiol vs. ABPPk + H_2_O_2_: ^&^*P* < 0.05, *n* = 3.

## Abbreviations

ABPPk: *Achyranthes bidentata* polypeptide fraction k
DAPI: 4’,6-Diamidino-2-Phenylindole
GAPDH: glyceraldehyde-3-phosphate dehydrogenase
GSH: glutathione
H_2_O_2_: hydrogen peroxide
HPLC: high-performance liquid chromatography
HRP: horseradish peroxidase
LDH: lactic dehydrogenase
MDA: malondialdehyde
MTT: 3-(4,5-dimethylthiazol-2-yl)-2,5-diphenyltetrazolium bromide
SOD: superoxide dismutase
