# Supplementary figures and images for: Achyranthes bidentata Polypeptide Protects Schwann Cells From Apoptosis in Hydrogen Peroxide-Induced Oxidative Stress
Source: Front Neurosci. 2018 Nov 30;12:868. doi: 10.3389/fnins.2018.00868 (PMC6284036; doi:10.3389/fnins.2018.00868)

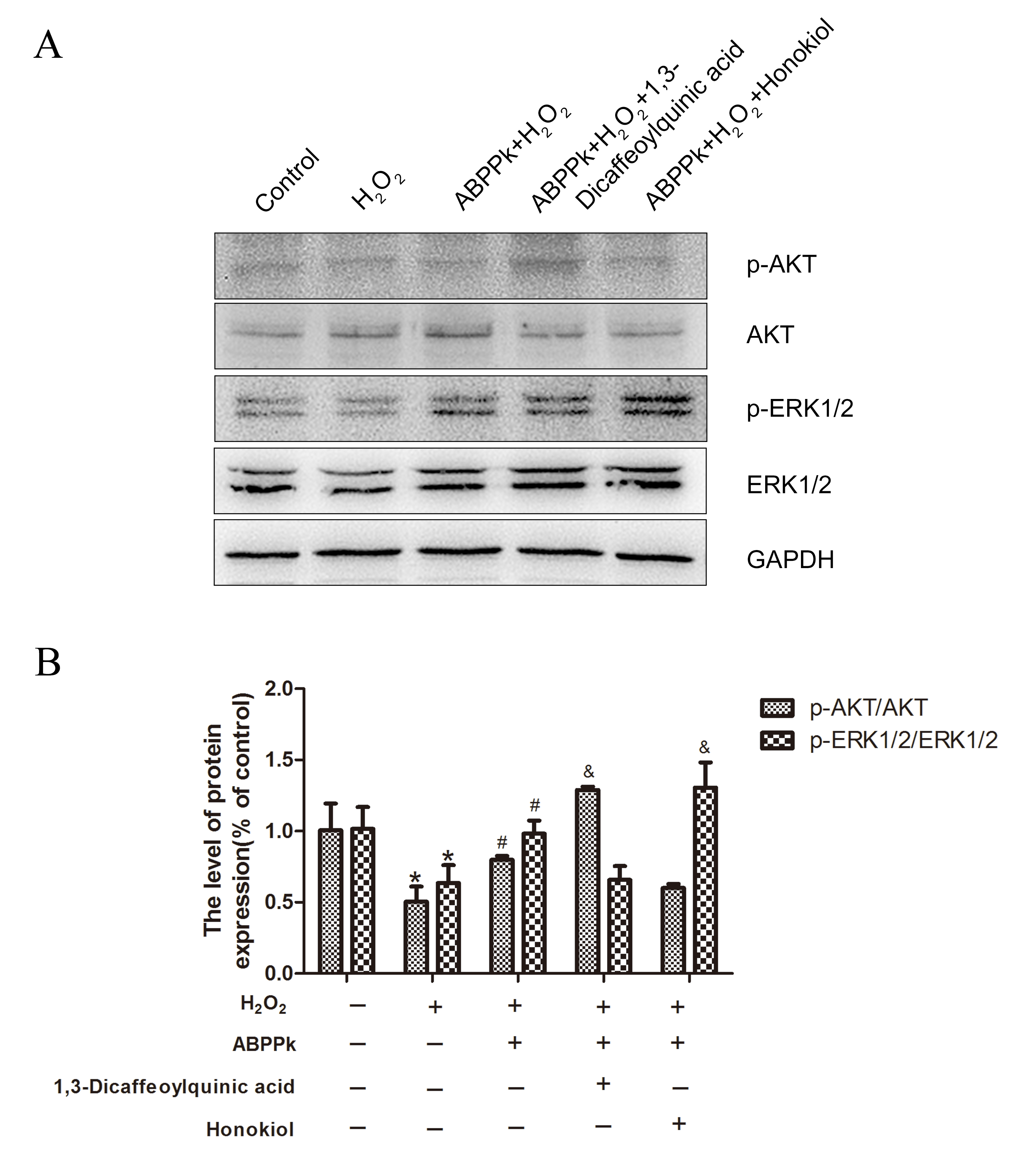

Supplement: FIGURE S1 — Effects of the PI3K/AKT agonists 1,3-Dicaffeoylquinic acid and ERK1/2 agonists Honokiol on ABPPk induced attenuation of oxidative damage. (A) SCs were treated with the agonists 1,3-Dicaffeoylquinic acid (10 μM) and Honokiol (10 μM) for 1 h before the treatment of H2O2 (400 μM) and ABPPk (0.5 μg/ml) for 24 h. (B) Densitometric analyses illustrates the results of p-AKT/AKT and p-ERK/ERK. H2O2 vs control: ∗P < 0.05; ABPPk + H2O2 vs H2O2: #P < 0.05; ABPPk + H2O2 + 1,3-Dicaffeoylquinic acid or ABPPk + H2O2 + Honokiol vs. ABPPk + H2O2: &P < 0.05, n = 3. [file Image_1.TIF]
